# Genetic Ablation of Nrf2/Antioxidant Response Pathway in Alexander Disease Mice Reduces Hippocampal Gliosis but Does Not Impact Survival

**DOI:** 10.1371/journal.pone.0037304

**Published:** 2012-05-31

**Authors:** Tracy L. Hagemann, Emily M. Jobe, Albee Messing

**Affiliations:** 1 Waisman Center, University of Wisconsin, Madison, Wisconsin, United States of America; 2 Graduate Program in Cellular and Molecular Biology, University of Wisconsin, Madison, Wisconsin, United States of America; 3 Department of Comparative Biosciences, University of Wisconsin, Madison, Wisconsin, United States of America; University of Durham, United Kingdom

## Abstract

In Alexander disease (AxD) the presence of mutant glial fibrillary acidic protein (GFAP), the major intermediate filament of astrocytes, triggers protein aggregation, with marked induction of a stress response mediated by the transcription factor, Nrf2. To clarify the role of Nrf2 in AxD, we have crossed Gfap mutant and transgenic mouse models into an Nrf2 null background. Deletion of Nrf2 eliminates the phase II stress response normally present in mouse models of AxD, but causes no change in body weight or lifespan, even in a severe lethal model. AxD astrocytes without Nrf2 retain features of reactivity, such as expression of the endothelin-B receptor, but have lower Gfap levels, a decrease in p62 protein and reduced iron accumulation, particularly in hippocampus. Microglial activation, indicated by Iba1 expression, is also diminished. Although the Nrf2 response is generally considered beneficial, these results show that in the context of AxD, loss of the antioxidant pathway has no obvious negative effects, while actually decreasing Gfap accumulation and pathology. Given the attention Nrf2 is receiving as a potential therapeutic target in AxD and other neurodegenerative diseases, it will be interesting to see whether induction of Nrf2, beyond the endogenous response, is beneficial or not in these same models.

## Introduction

Alexander disease (AxD) in its most common form is a fatal neurodegenerative disorder, typically affecting young children with early onset. The pathologic hallmark is widespread deposition of inclusion bodies called Rosenthal fibers in sub-pial, peri-vascular, and peri-ventricular astrocytes, and consisting of aggregated GFAP and other intermediate filament proteins, plectin, ubiquitin, small heat shock proteins, and likely other unidentified proteins [1]–[3]. Nearly all Alexander patients, including those with late onset juvenile or adult forms of the disease, carry heterozygous mutations within the coding region of the gene for GFAP [4], [5]. These mutations predict expression of abnormal GFAPs which act in a dominant gain-of-function fashion [6].

Although AxD is genetically homogenous, there is considerable variability in severity of disease even among individuals carrying identical mutations [7]. The common R79 and R239 mutations cause both infantile and juvenile onset forms of the disease, and R416W causes all three forms of the disorder, including adult [5]. In some cases even individuals within the same family, carrying the same mutation, show variability with mixed juvenile-adult presentations, as has been found for D78E [8], S247P, and D417A [9], or may be completely asymptomatic as with L331P [10]. Perhaps the rare mutations show variable penetrance, or there are genetic modifiers that influence the course of disease.

To facilitate mechanistic studies of AxD pathogenesis, and provide animal models suitable for testing potential therapies, we have generated knock-in lines of mice carrying the most common GFAP mutations found in human AxD (equivalent to R79H and R239H), and found that expression of mutant Gfap induces formation of Rosenthal fibers, increases susceptibility to kainate induced seizures [11], alters adult neurogenesis and leads to deficits in learning (T.L. Hagemann, et al., manuscript in preparation). Altering Gfap expression either by production of mutant Gfap or simple over-expression induces multiple stress pathways [11]–[15] that suggest specific strategies for therapy [16]. In addition, expressing mutant Gfap in the context of elevated wild-type GFAP intensifies this stress response and results in terminal seizures [11].

Nrf2 (otherwise known as Nfe2l2: nuclear factor, erythroid derived 2, like 2) is a transcription factor that binds to a short antioxidant response element (ARE) found in the promoters of a number of detoxification genes including those involved in redox homeostasis, glutathione turnover, and iron metabolism. As a group, these genes are up-regulated in response to oxidative stress. Previously we have found increased expression of Nrf2-regulated target genes, such as Nqo1, in both human brain samples from Alexander patients as well as in GFAP over-expressing transgenic mice [12]. One mechanism by which Nrf2 might be elevated is impairment of the ubiquitin-proteasome system [17], a common feature of protein aggregation disorders that is found in AxD as well [13], [18]. Nrf2 is regulated through two degradation domains, Neh2 and Neh6, by association with E3 ubiquitin ligase adaptor proteins Keap1 and β-TrCP respectively. Keap1, in response to oxidative stress, undergoes a conformational change that interferes with targeting Nrf2 for ubiquitination, and therefore proteasomal degradation [19]. In contrast, recognition of the Neh6 domain by β-TrCP is redox independent and mediated by serine/threonine glycogen synthase kinase, GSK-3β [20], [21]. Under normal conditions, GSK-3β is relatively inactive, and Keap1 is responsible for directing Nrf2 degradation. Under conditions of stress, Nrf2 is activated by release from Keap1, however phosphorylation by GSK-3β can again tag Nrf2 for ubiquitination and degradation. Proteasome dysfunction and accumulation of misfolded proteins as observed in AxD [18] would not only lead to oxidative inactivation of Keap1, but also prohibit effective clearing of ubiquitinated Nrf2.

As with many aspects of the cellular stress response, the Nrf2 pathway may lead to both beneficial and harmful effects. Nrf2 expression in the mouse appears overall to provide a protective response in the CNS. For instance, Nrf2-null animals show increased susceptibility to ischemic injury in the brain [22], and enhancing Nrf2 expression in astrocytes confers protection against both toxic and genetic insults to astrocytes and to neighboring neurons [23]–[28]. In contrast, constitutive activation of Nrf2 leads to a lethal phenotype in Keap1 knockout mice [29], and a recent report shows harmful effects for sustained Nrf2 activation in a transgenic model of protein aggregation cardiomyopathy [30]. To gain insight into the role of Nrf2-regulated genes in the pathogenesis of AxD, we have examined the consequences of placing the Alexander models in an Nrf2-null background.

In this report, we show that loss of Nrf2 in our AxD models has surprisingly little effect on the overall health of the animals. Rosenthal fiber distribution remains the same, despite reduced expression of the Nrf2 regulated ubiquitin binding protein p62, and both astrocytes and microglia are still reactive. However, we observe that stress response genes unrelated to Nrf2 are down regulated, including αB-crystallin (Cryab), ceruloplasmin (Cp), and Gfap itself. In addition iron accumulation in astrocytes is also reduced, suggesting that glia are less reactive and that the Nrf2 induced stress response is in part promoting pathology rather than preventing it.

## Materials and Methods

### Ethics Statement and Animal Care and Use

**Figure 1:**
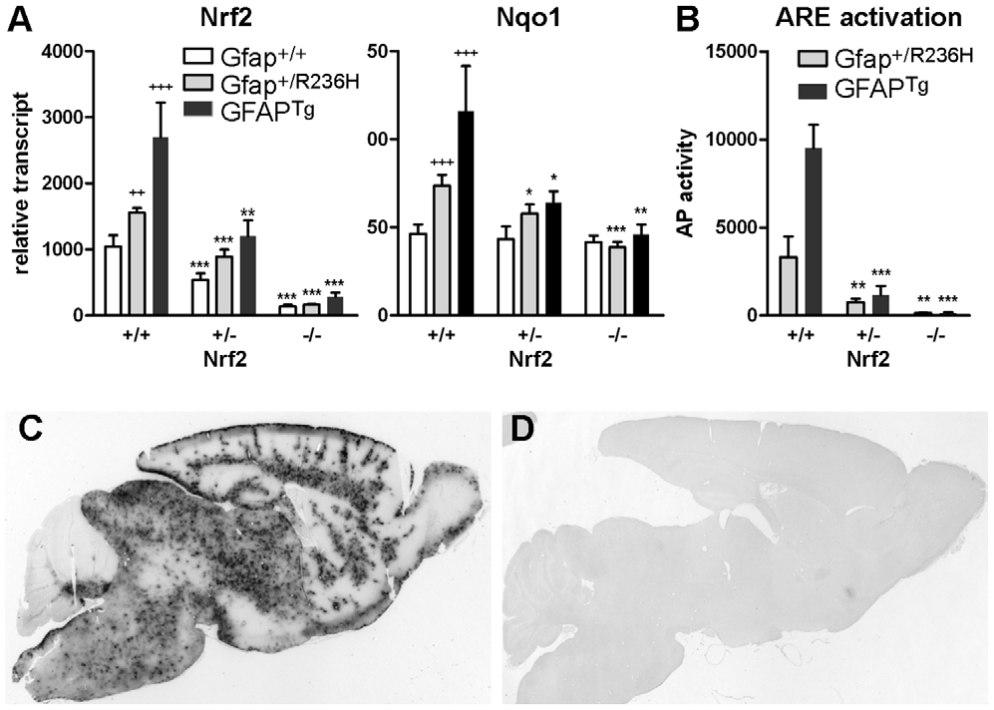
Knockout of Nrf2 in Gfap mutant or transgenic mice eliminates ARE (antioxidant response element) activation. (**A**) Quantitation of brain transcripts from Nrf2 and its target gene Nqo1 for both GFAP^Tg^ and Gfap^+/R236H^ mice (3 mos of age) shows reduced expression in Nrf2^+/−^ background and further reduction in Nrf2^−/−^. (**C, D**) Histochemical staining for alkaline phosphatase activity in GFAP^Tg^ mice crossed with an ARE-alkaline phosphatase reporter line shows wide-spread activation of Nrf2 (**C**), whereas GFAP^Tg^/Nrf2^−/−^ mice show virtually no ARE-reporter activity (**D**; 6 wks of age). (**B**) Quantification of reporter activity in brains from either GFAP^Tg^ or GFAP^+/R236H^ mice shows a marked reduction in Nrf2^+/+^ vs. Nrf2^+/−^ mice and virtually no detectable activity in Nrf2^−/−^ animals (3 mos of age). Error bars =  standard deviation. For Gfap^+/+^ versus Gfap^+/R236H^ or GFAP^Tg^ comparisons (all Nrf2^+/+^), significance is indicated with a (^+^) plus sign. For Nrf2^+/+^ vs Nrf2^+/−^ or ^−/−^ comparisons, significance is indicated by an (*) asterisk (unpaired t-test: ^+ or^ *p<0.05; ^++ or^ **p<0.01; ^+++ or^ ***p<0.001, n≥3).

This study was approved by the Animal Care and Use Committee for the Graduate School at the University of Wisconsin, Madison. All animals were cared for and used in accordance with standards set by the Committee. Gfap^+/R236H^ point mutant knock-in mice were maintained as heterozygotes [11] either as a mixed strain (early generation backcross into 129S6 from FVB/N, Figure 1) or C57BL/6J (>10 generations). GFAP^Tg^ [31] and ARE-hPAP transgenic mice [32] were maintained as hemizygotes in FVB/N, and Nrf2 knockout mice [33] were bred as homozygotes in strain C57BL/6J. Gfap^+/R236H^/Nrf2^−/−^ and GFAP^Tg^/Nrf2^−/−^ mice are F2 offspring derived from cross mating into the Nrf2 null background.

### Quantitative PCR

Mice were euthanized by CO_2_ asphyxiation, dissected, tissues collected and frozen in liquid nitrogen. For initial analysis of Nrf2 and Nqo1 transcripts, total RNA was extracted with Trizol (Invitrogen, Carlsbad, CA) from half brains bisected at the midline (Figure 1). Subsequent analysis was performed with RNA extracted from either olfactory bulb or hippocampus. Complementary DNA was synthesized from 1 µg RNA with Superscript II (Invitrogen) and real-time PCR performed with SYBR Green Master Mix on an ABI 7500 sequence detection system (Applied Biosystems, Foster City, CA) as previously described [11]. Primer sets for quantifying transcripts are included in Table 1.

**Table 1:** Primer sets for quantitative PCR.

Probe	Accession No.	Forward Primer	Reverse Primer
Nfe2l2	NM_010902	GATCCGCCAGCTACTCCCAGGTTG	CAGGGCAAGCGACTCATGGTCATC
Nqo1	NM_008706	CGGTATTACGATCCTCCCTCAACA	AGCCTCTACAGCAGCCTCCTTCAT
Cryab	M63170	CCAGTTCTTCGGAGAGCACCT	CTGTCCTTCTCCAAACGCATC
Cp	NM_007752	CGAGCCGAAGAAGACGAGCACTT	TCACCCCATGGGCATGTATTGAAT
Gfap	NM_010277	CAACGTTAAGCTAGCCCTGGACAT	CTCACCATCCCGCATCTCCACAGT
Fth1	NM_010239	GTGCGCCAGAACTACCACCAGGAC	GCAAAGTTCTTCAGAGCCACATCATC
18SrRNA	X00686	CGCCGCTAGAGGTGAAATTCT	CGAACCTCCGACTTTCGTTCT

### Immunoblotting and Western Analysis

Tissues were collected as above and protein extracted by homogenization in RIPA lysis buffer (20 mM Tris-HCl pH 7.5; 150 mM NaCl; 1 mM EDTA; 1% Triton-X-100; 0.5% sodium deoxycholate; 0.1% SDS; 1 mM Pefabloc SC (Sigma); and Complete Protease Inhibitor Cocktail (Roche Applied Science, Indianapolis, IN)). Lysates were centrifuged 17,000 g at 4°C for 20 min, supernatant collected, and protein quantified with the BCA assay (Thermo Scientific/Pierce Biotechnology, Rockford, IL). Protein samples (15 µg) were electrophoresed on 10 or 15% polyacrylamide gels, transferred to Immobilon-FL membrane (Millipore), and blocked with Odyssey blocking buffer (Li-Cor, Lincoln, NE). Immunoblots were incubated with primary antibody diluted in TBS with 0.05% Tween20 overnight at 4°C at the following dilutions: 1∶1000 anti-Iba1 (Wako 019-19741, Osaka, Japan), 1∶2000 anti-SQSTM1/p62 (Abnova H00008878-M01, Taipei Taiwan), 1∶2000 goat anti-FTL (Abnova PAB11455) 1∶1000 rabbit anti-FTH1 (Cell Signaling #3998, Beverly, MA), 1∶5000 mouse anti-GAPDH (Fitzgerald Industries 10R-G109A, Acton, MA). Membranes were washed with TBS/Tween20 before incubating with secondary antibodies: AlexaFluor 680 goat anti-mouse (Invitrogen), DyLight 800 goat anti-rabbit (Thermo Scientific) or IRDye 800CW donkey anti-goat IgG (Li-Cor) 1∶10,000 in TBS/Tween20 for 2 hrs. Immunoblots were analyzed with an Odyssey Infrared Imaging System (Li-Cor), and signal intensity for proteins of interest normalized to that of Gapdh.

### ELISA for Gfap

Gfap quantification by ELISA was carried out as previously described [11], [34] with some modifications. Briefly, samples were homogenized in total lysis buffer (2% SDS/50 mM Tris-HCl pH7.4/5 mM EDTA/1 mM Pefabloc SC/Complete Protease Inhibitor Cocktail; 1∶20 weight to volume), and boiled for 15 min. Protein was quantified with the BCA assay and diluted to approximately 0.5 µg/ml. Microtiter plates were coated with a monoclonal antibody cocktail against GFAP (SMI-26, Covance, Princeton, NJ) diluted 1∶1000 in PBS overnight at 4°C. Nonspecific binding of the capture antibody was blocked with 5% milk in PBS (BLOTTO, room temperature for 2 hrs) before applying purified GFAP standard (RDI Division of Fitzgerald Industries Intl, Concord, MA) or approximately 50 ng of protein sample diluted in 0.05% Tween 20 in PBS (PBS/Tw20) with 1% BSA. After incubating 2 hrs at room temperature, plates were rinsed with PBS/Tw20 and rabbit polyclonal anti-GFAP applied (DAKO Z334 at 0.2 µg/ml BLOTTO) overnight at 4°C for detection. Plates were rinsed with PBS/Tw20 and incubated with HRP conjugated goat anti-rabbit secondary (1∶10,000 in BLOTTO, Sigma A6154) for 2 hrs at room temperature. After a final rinse SuperSignal ELISA Femto Chemiluminescent Substrate (Thermo Scientific/Pierce Biotechnology) was added and the reaction quantified with a Turner Biosystems GloRunner Luminometer (Sunnyvale, CA).

### Immunostaining

For immunohistochemistry, animals were anesthetized and perfused transcardially with 10 ml phosphate buffered saline (PBS) followed by 50 ml 4% paraformaldehyde in PBS, pH 7.4; brains were removed, fixed overnight and then cryoprotected in 30% sucrose. Tissues were frozen in OTC and 30 µm sagittal sections were cut on a sliding microtome. Endogenous peroxidases were blocked and tissues permeabilized as floating sections in 0.15% hydrogen peroxide with 0.3% Triton-X-100 in PBS for 10 min, followed by 5% normal donkey serum with 0.3% Triton-X-100 in PBS for 2 h. Primary antibodies were diluted in PBS with 1.0% BSA and 0.3% Triton-X-100 [1∶500 mouse anti-GFAP, Millipore MAB3402 (Billerica, MA); 1∶500 rabbit anti-Iba1, Wako 019–19741 (Osaka, Japan); 1∶200 mouse anti-SQSTM1/p62, H00008878-M01, Abnova (Taipei, Taiwan)] and tissues incubated overnight at 4°C. Secondary antibodies were diluted in the same [1∶200 biotinylated anti-mouse or anti-rabbit, ABC kit, Vector Labs (Burlingame, CA)] and incubated for 2 h. Sections were incubated with ABC solution for 30 min followed by Vector SG chromogen for 5 min. Between stages, sections were washed thoroughly in PBS, and finally mounted on slides, dried and coverslipped with Permount mounting media. Images were taken with a Nikon Microphot equipped with a SPOT digital camera (Diagnostic Instruments, Sterling Heights, MI).

For immunofluorescence, sections were prepared as described above, blocked and permeabilized in 5% normal donkey serum with 0.3% Triton-X-100 in PBS for 2 h. Primary antibodies were diluted in PBS with 1% BSA and 0.3% Triton-X-100 [1∶100 rat anti-Mac1, CD11b, Pharmingen (Franklin Lakes, NJ); 1∶1,000 rabbit anti-Ferritin, Sigma F6136 (St Louis, MO); 1∶200 mouse anti-SQSTM1, Abnova H00008878-M01 (Taipei City, Taiwan); 1∶100 rabbit anti-endothelin-B receptor, Alomone Labs AER-002 (Jerusalem, Israel); 1∶1000 rabbit anti-GFAP, Dako Z0334 (Carpinteria, CA) or 1∶500 mouse anti-GFAP, Millipore MAB3402] and incubated at 4°C overnight; secondary antibodies were diluted in the same (1∶50 FITC conjugated donkey anti-rat IgG, Sigma; 1∶500 AlexaFluor-546 or -488 donkey anti-rabbit or-mouse IgG, Invitrogen) and incubated for 2 h. Sections were mounted on slides and coverslipped with VectaSheild mounting media (Vector Labs) and images taken with a Nikon C1 scanning confocal microscope.

### Iron and Alkaline Phosphatase Histochemistry

To detect differences in iron distribution or activation of the ARE-hPAP reporter within the brain, tissues were collected and processed as previously described [11]: with a modified Perl’s stain to detect iron [35] or with the substrate BCIP to detect alkaline phosphatase activity.

### Alkaline Phosphatase Reporter Assay

To quantify alkaline phosphatase activity resulting from activation of the ARE-hPAP reporter, tissues were homogenized in CHAPS lysis buffer (50 mM Tris-HCl pH 7.5/5 mM MgCl_2_/100 mM NaCl/4% CHAPS) and centrifuged at 17,000 g for 20 min at 4°C. Supernatants were collected and protein quantified with the BCA assay. Samples were diluted to 100 µg/ml, 25 µl added to 75 µl 200 mM diethanolamine (DEA) in a microtiter plate, and heated for 20 min at 65°C to inactivate endogenous alkaline phosphatase activity. At room temperature, 100 µl chemiluminescent substrate CSPD with Emerald enhancer (2x; Tropix- Applied Biosystems, Bedford, MA) in 5 mM MgCl_2_/150 mM DEA was added to each well, and incubated at room temperature for 20 min. Enzyme activity was quantified with a GloRunner Microplate Luminometer.

## Results

Mouse models of Alexander disease were crossed into an Nrf2^−/−^ null background to determine the role of the phase II stress response activated by this transcription factor in AxD pathology. Nrf2 knockout mice have a targeted deletion of part of exon 4 and all of exon 5. This includes the cap-n-collar, DNA binding, and leucine zipper domains [33]. The AxD models include two lines of mice: 1) an R236H point mutation knock-in of the endogenous mouse Gfap gene (Gfap^+/R236H^), mimicking a common mutation in humans with the disease (R239H), and 2) a transgenic model over-expressing wild-type human GFAP (GFAP^Tg^). Both lines present Rosenthal fibers (RF), the hallmark pathology of AxD in astrocytes, and a decrease in body weight, with GFAP^Tg^ mice being the more severely affected of the two models. The phase II stress response, as measured with a reporter line expressing alkaline phosphatase (hPAP) under the control of an antioxidant response element (ARE), and confirmed by quantifying a number of transcripts from genes regulated by Nrf2, is elevated in both lines with a pattern of activation following the distribution of RF throughout the CNS [11], [12].

### Loss of Nrf2 Activated Stress Response does not Change the General Phenotype of AxD Mice

Both Gfap^+/R236H^ and GFAP^Tg^ mice show a decrease in body weight compared to wild-type littermates. After crossing either line into an Nrf2 null background, no further differences in weight or lifespan up to 6 months were observed (data not shown). To confirm the loss of Nrf2 expression in these mice, brain transcripts were quantified by real-time PCR with a probe recognizing coding sequence from exons 2−3. Even though non-functional transcripts from the mutant allele include sequences from exons 2 and 3, heterozygous knockout mice showed approximately 50% less Nrf2 transcript, whereas homozygous knockouts show a 90% reduction, indicating that the targeted transcript is unstable in agreement with the original report [33] (Figure 1A). Expression of Nqo1, a notable target of Nrf2 activation, is significantly elevated in both Gfap^+/R236H^ and GFAP^Tg^ mice [11], [12], but Nqo1 expression is reduced to that of wild-type mice in both Gfap^+/R236H^/Nrf2^−/−^ and GFAP^Tg^/Nrf2^−/−^ animals (Figure 1A).

The same genetic combinations were crossed with the ARE-hPAP reporter line expressing alkaline phosphatase regulated by the antioxidant response element (ARE) from the rat Nqo1 gene. Expression of the reporter was reduced by more than half in Nrf2^+/−^ heterozygotes, and completely absent in Nrf2^−/−^ animals (Figure 1B, C, D).

Most experiments were performed with both Gfap^+/R236H^ and GFAP^Tg^ models, but for this report, we focus on the Gfap^+/R236H^ point mutant since it more closely replicates the molecular defect of the human disorder. Although the two models differ in severity, both show a similar response to the loss of Nrf2.

### Astrocyte Reactivity is Reduced in AxD Models without Nrf2 Induced Stress Response

**Figure 2:**
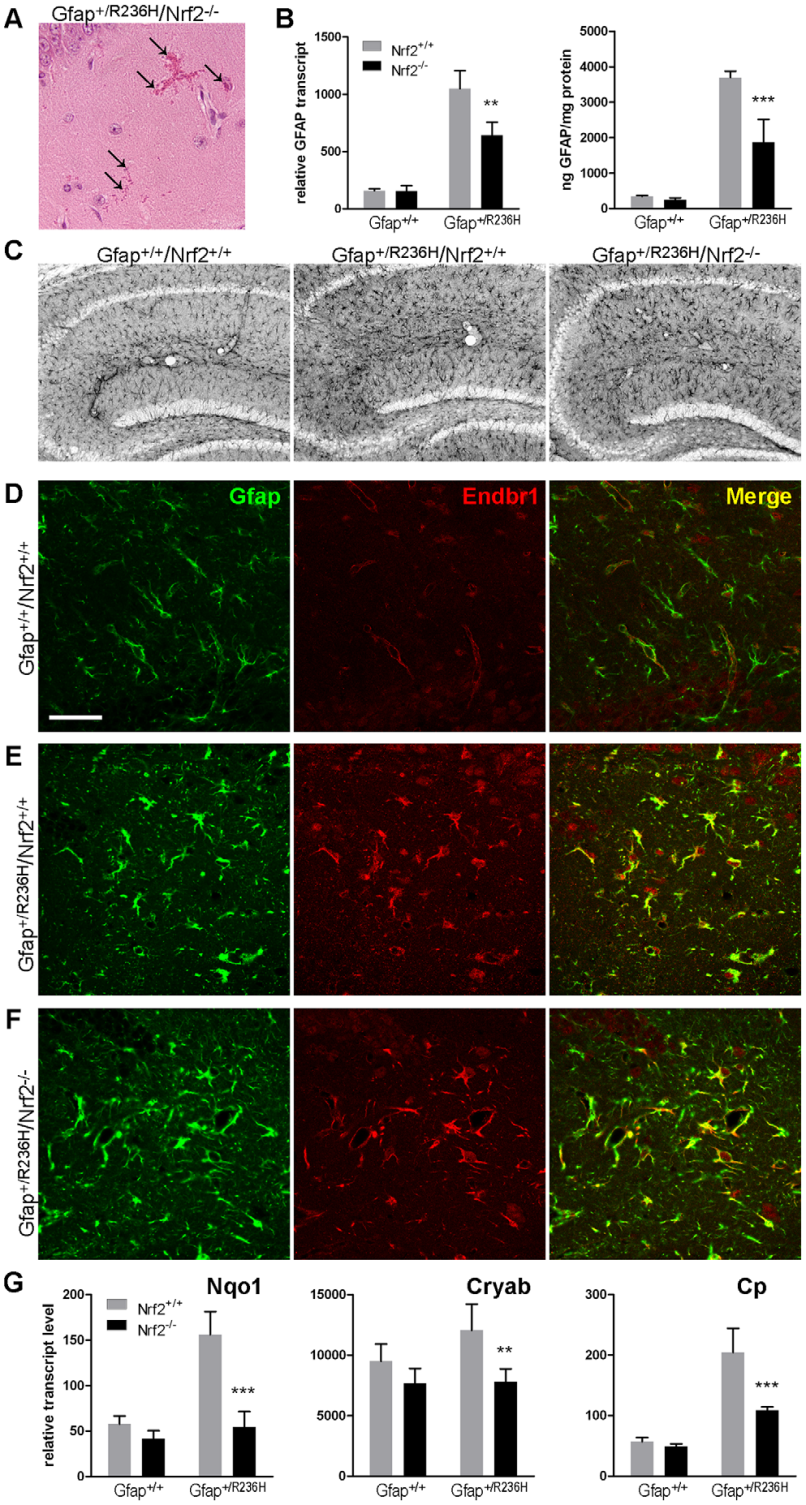
Astrocytes from Gfap^ +/R236H^/Nrf2^−/−^ mice exhibit Rosenthal fibers and appear reactive, but Gfap and stress markers are reduced. (A) Rosenthal fibers are apparent as eosinophilic aggregates in H&E stained tissue (arrows, hippocampus CA1 shown) in Gfap^+/R236H^/Nrf2^−/−^ mice. (B) Quantitation of Gfap transcript by QPCR and protein by ELISA shows reduced Gfap expression in Gfap^+/R236H^/Nrf2^−/−^ versus Gfap^+/R236H^/Nrf2^+/+^ mice in hippocampus (8 wks of age, n≥4, t-test **p>0.01, ***p>0.001 comparing Gfap^+/R236H^/Nrf2^+/+^ with Gfap^+/R236H^/Nrf2^−/−^). (C) Compared with wild-type Gfap^+/+^/Nrf2^+/+^ mice, Gfap immunohistochemistry in hippocampus (gray stain, 3 mos of age) shows hypertrophic reactive astrocytes in to Gfap^+/R236H^/Nrf2^+/+^ and Gfap^+/R236H^/Nrf2^−/−^ mice. (D-F) An alternative marker of reactive astrocytes, the endothelin-B receptor (Endbr1, red), is expressed in astrocytes (Gfap, green) from both Gfap^+/R236H^/Nrf2^+/+^ (E) and Gfap^+/R236H^/Nrf2^−/−^ mice (F), compared with wild-type Gfap^+/+^/Nrf2^+/+^ (D) which show Endbr1 expression predominantly associated with blood vessels (hilus, 8 wks of age). Scale bar = 50 µm (shown in D, applies to all confocal images). (G) Hippocampal expression of stress related genes, including Nqo1, Cryab, and Cp, is reduced in Gfap^+/R236H^ mice with the loss of Nrf2 (8 wks). Error bars indicate standard deviation (t-test **p<0.01, ***p<0.001; n = 4).

To determine whether the lack of Nrf2 affects astrocyte reactivity, we analyzed the response in hippocampus and olfactory bulb, regions that show high levels of RF, Gfap expression and Nrf2 induced stress response in Gfap^+/R236H^ mice. Nrf2 null AxD mice demonstrate RFs in these regions, similar to their Nrf2^+/+^ counterparts (Figure 2A, hippocampus shown). Gfap transcripts and protein were lower in hippocampus from Gfap^+/R236H^/Nrf2^−/−^ mice, but still above that of Gfap^+/+^ mice (Figure 2B), and astrocytes retain a hypertrophied morphology indicative of reactive glia (Figure 2C). The endothelin-B receptor (Endbr1), an additional marker of gliosis, is expressed by astrocytes in both Gfap^+/R236H^/Nrf2^+/+^ and Gfap^+/R236H^/Nrf2^−/−^ mice (Figure 2E,F), compared to wild-type Gfap^+/+^/Nrf2^+/+^ mice where Endbr1 localizes mostly with the vasculature (Figure 2D).

As predicted from our analysis of brain transcripts (Figure 1A) Nqo1 levels in Gfap^+/R236H^/Nrf2^−/−^ hippocampus were reduced to that of wild-type animals (Figure 2G). However, transcripts for αB-crystallin (Cryab), a small heat shock protein and component of RF, and ceruloplasmin (Cp), an astrocyte expressed ferroxidase and acute phase response protein, were also reduced in hippocampus (Figure 2G), and neither are known to be regulated by Nrf2. In olfactory bulb from Gfap^+/R236H^/Nrf2^−/−^ mice, Nqo1 transcripts were reduced to wild-type levels, but neither Gfap nor Cryab showed significant reductions. Ceruloplasmin however was reduced by 24% between Gfap^+/R236H^/Nrf2^+/+^ and Gfap^+/R236H^/Nrf2^−/−^ mice (data not shown).

### Reduced Activation of Microglia

**Figure 3:**
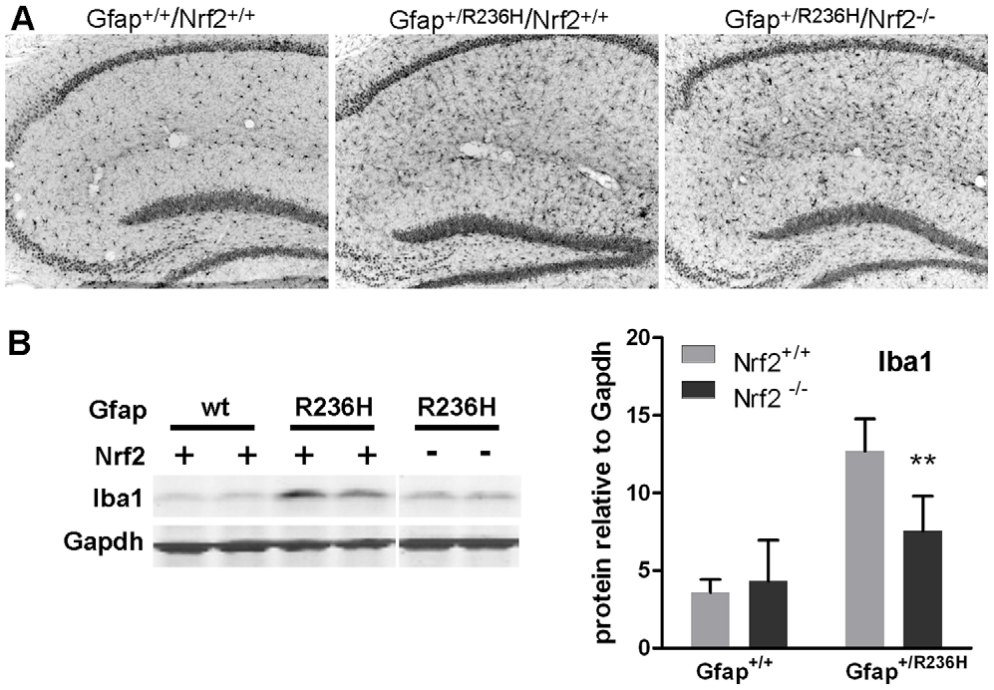
Microglia are less reactive in Gfap^+/R236H^ mice without Nrf2. (**A**) Compared to wild-type mice, Gfap mutant mice show elevated Iba1 immunoreactivity in hippocampus either with or without Nrf2. (**B**) Western analysis of hippocampal protein shows reduced Iba1 expression in Gfap^+/R236H^/Nrf2^−/−^ compared with Gfap^+/R236H^/Nrf2^+/+^ mice (8 wks), although levels remain elevated above wild-type (**p<0.01, unpaired t-test comparing Gfap^+/R236H^/Nrf2^+/+^ with Gfap^+/R236H^/Nrf2^−/−^ mice, n = 4). Lanes 5 and 6 for Nrf2^−/−^ samples are from a different region of the same gel as lanes 1–4 (Nrf2^+/+^ samples).

Although AxD is a primary disorder of astrocytes, microglia react to the resulting pathology. Histological analysis of Gfap mutants shows an increase in hyper-ramified Iba1 positive glia in both Nrf2^+/+^ and Nrf2^−/−^ mice (Figure 3A). Western analysis shows reduced expression of Iba1 in both hippocampus and olfactory bulb from Nrf2^−/−^ mice, suggesting a reduced microglial response (Figure 3B: hippocampus shown). This change could be cell autonomous due to the loss of Nrf2 in microglia, or reflect a decreased response to less reactive astrocytes.

### p62 is Present in Rosenthal Fibers, and Reduced by Nrf2 Knockout

**Figure 4:**
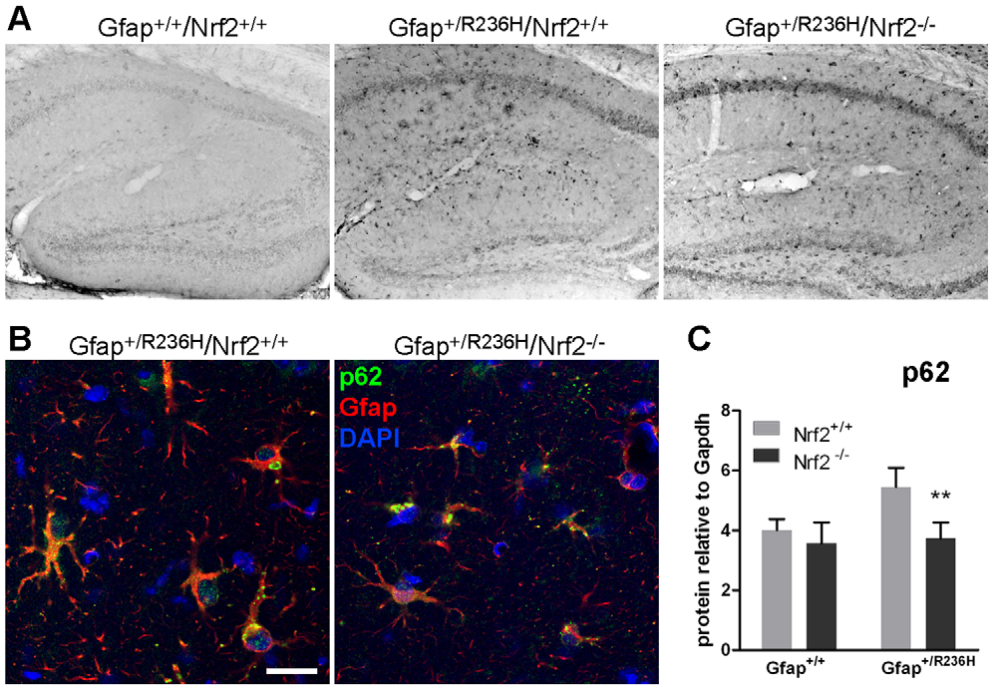
p62 in Gfap^+/R236H^ mice colocalizes with Gfap aggregates and is reduced with loss of Nrf2. (**A**) p62 immunohistochemistry in hippocampus from Gfap^+/R236H^/Nrf2^+/+^ mice shows astrocytic cells that are not apparent in Gfap^+/+^ mice. Gfap^+/R236H^/Nrf2^−/−^ mice show similarly stained cells (3 mos of age). (**B**) Confocal microscopy with immunofluorescence for p62 (green) shows colocalization with Gfap aggregates (red) in both Nrf2^+/+^ and Nrf2^−/−^ Gfap^+/R236H^ mice (8 wks of age). (**C**) Western blot analysis of hippocampal protein shows increased p62 in Gfap mutant mice compared to wild-type, with Gfap^+/R236H^/Nrf2^−/−^ mice having levels reduced to that of wild-type mice (8 wks of age, n≥4, t-test **p>0.01, comparing Gfap^+/R236H^/Nrf2^+/+^ with Gfap^+/R236H^/Nrf2^−/−^). Scale bar = 25 µm (shown in **B**, applies to both confocal images).

Sequestosome/p62 binds both ubiquitin and LC3 and functions to target ubiquitinated proteins for autophagy. p62 is also transcriptionally regulated by Nrf2. Immunostaining shows increased p62 reactivity in Gfap mutant compared to wild-type astrocytes (Figure 4A) and colocalization with some Gfap aggregates (Figure 4B). p62 positive aggregates are also apparent in Gfap^+/R236H^/Nrf2^−/−^ mice, but histological staining appears less intense (Figure 4A,B) and western analysis shows decreased levels of p62 in hippocampus (Figure 4C), but not olfactory bulb (data not shown).

### Iron Accumulation in GFAP Mutant Mice and Ferritin Regulation by Nrf2

**Figure 5:**
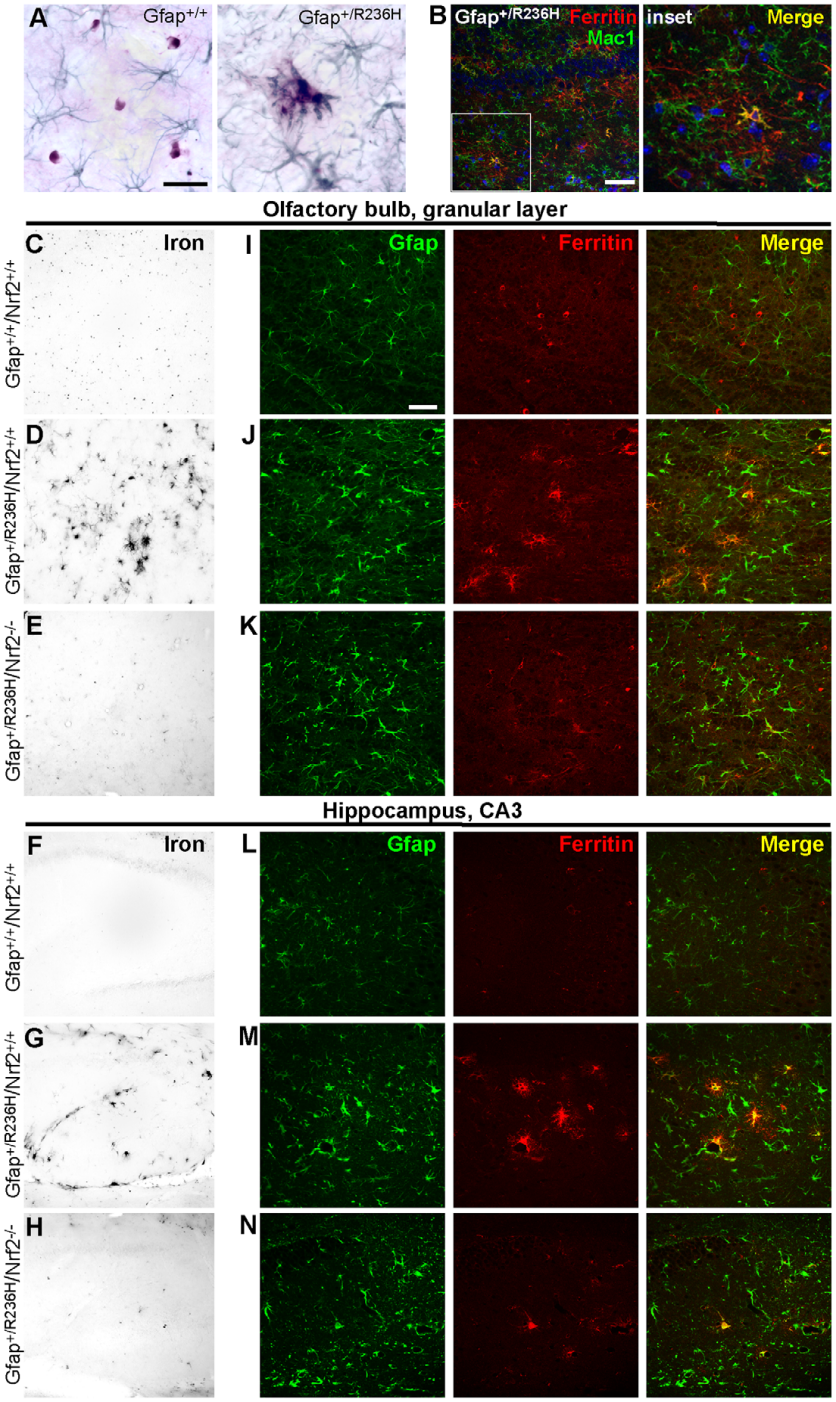
Iron accumulation in astrocytes from Gfap mutants is reduced with loss of Nrf2. (**A**) Modified Perl’s stain shows iron in small round cell bodies (brown) with oligodendrocyte morphology separate from Gfap immunostained astrocytes (blue/green) in Gfap^+/+^ olfactory bulb, whereas iron staining is prominent in astrocytes with reactive morphology in Gfap^+/R236H^ mice and in some cases appears to localize with Gfap aggregates (3 mos of age). (**B**) Ferritin immunofluorescence shows occasional colocalization with Mac1 (hippocampus, 3 mos of age) suggesting some iron storage in microglia. (**C-H**) Iron histochemistry shows accumulation in astrocytes of both olfactory bulb (**C-E**) and hippocampus (**F-H**) in Gfap^+/R236H^ mice (**D, G**) compared with Gfap^+/+^ brains (**C, F**). Loss of Nrf2 reduces iron staining in Gfap^+/R236H^/Nrf2^−/−^ mice (**E, H**). (**I**-**N**) Immunostaining for ferritin (red) and Gfap (green) in olfactory bulb (**I-K**) and hippocampus (**L-N**) shows ferritin labeling in Gfap negative cells with oligodendrocyte morphology, especially in olfactory bulb (**I**) and sparse labeling in hippocampus (**L**) in wild-type mice at 8 wks of age. In Gfap^+/R236H^/Nrf2^+/+^ mice, ferritin colocalizes with Gfap in astrocytes in both olfactory bulb (**J**) and hippocampus (**M**). Gfap^+/R236H^/Nrf2^−/−^ mice (**K, N**) show an intermediate pattern with less staining in Gfap positive cells, and in olfactory bulb (**K**), cells that appear to be oligodendrocytes. Scale bars = 25 µm (**A**); 50 µm (**B,** **I**).

As we reported previously, brain iron accumulation is apparent in both GFAP^Tg^ and Gfap^+/R236H^ mice [11], [12]. Histochemical staining for iron usually delineates oligodendrocytes in the CNS, but in Gfap^+/R236H^ mice, immunostaining for Gfap shows iron accumulating mostly in astrocytes (Figure 5A). Nrf2 regulates both the heavy and light chain peptides of the iron storage protein ferritin, and Nrf2 knockout diminishes the accumulation of iron in astrocytes from AxD mice (Figure 5C, D, E, F, G, H). Immunofluorescence shows ferritin distribution following the pattern of iron storage in wild-type and Gfap^+/R236H^/Nrf2^+/+^ mice. In Gfap^+/+^/Nrf2^+/+^ mice ferritin immunostaining appears to highlight oligodendrocytes in olfactory bulb (Figure 5I) and is sparse in hippocampus (Figure 5L), whereas in Gfap^+/R236H^/Nrf2^+/+^ mice, ferritin colocalizes with Gfap in astrocytes (Figure 5J,M). Gfap^+/R236H^/Nrf2^−/−^ mice seem to have an intermediate phenotype with ferritin reactivity still apparent in astrocytes, but with less intensity (Figure 5K,N). Occasional microglia also show elevated ferritin in both Gfap^+/R236H^/Nrf2^+/+^ and Gfap^+/R236H^/Nrf2^−/−^ mice (Figure 5B: Gfap^+/R236H^/Nrf2^+/+^ shown).

**Figure 6:**
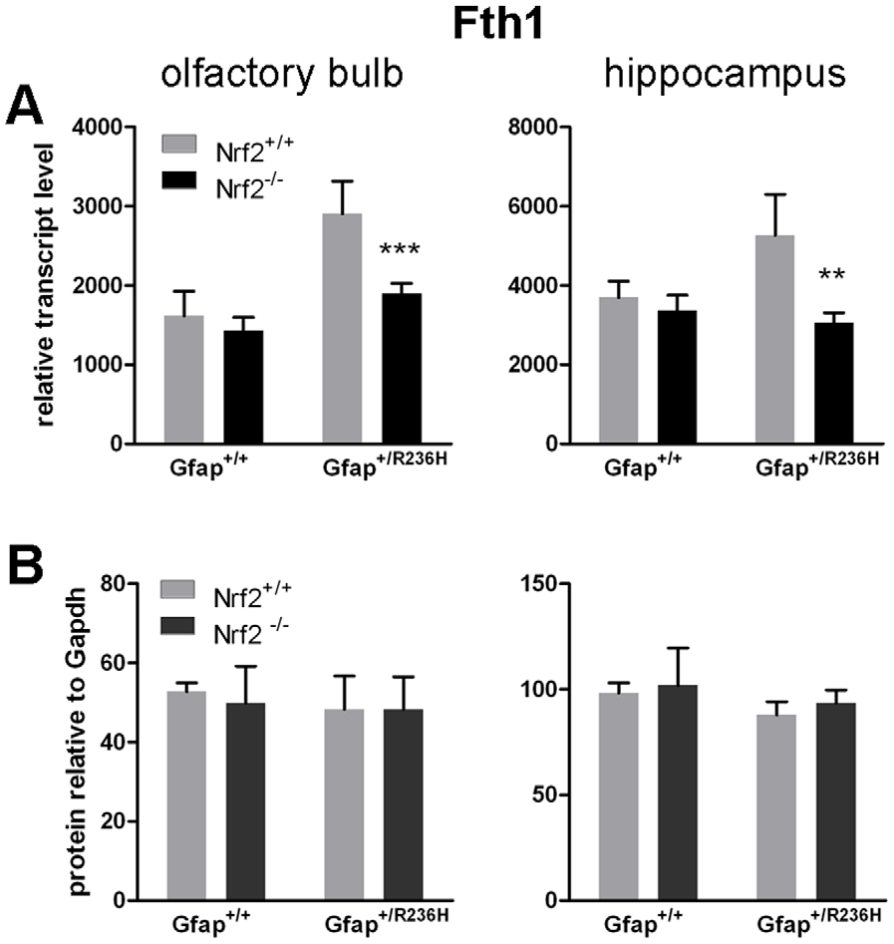
Ferritin transcripts and protein are differentially regulated. (**A**) Transcripts for the ferritin heavy chain gene Fth1 are elevated in both olfactory bulb and hippocampus from Gfap^+/R236H^ mice, and are reduced to wild-type levels with loss of Nrf2. (**B**) Fth1 protein levels, however remain unchanged in all genotypes. (8 wks of age, n = 4, t-test **p>0.01, ***p > 0.001 comparing Gfap^+/R236H^/Nrf2^+/+^ with Gfap^+/R236H^/Nrf2^−/−^).

On the transcriptional level, ferritin heavy chain (Fth1) expression is increased in Gfap^+/R236H^/Nrf2^+/+^ and reduced back to wild-type levels in Gfap^+/R236H^/Nrf2^−/−^ (Figure 6A), although ferritin light chain transcripts remain unchanged (Ftl1, data not shown). However ferritin is also regulated post-transcriptionally, and western analysis shows no change in heavy or light chain protein (Figure 6B; data not shown for Ftl1).

### Nrf2 Knockout does not Affect Survival in Gfap^+/R236H^/GFAP^Tg^ Mice, a Lethal Model of AxD

**Figure 7:**
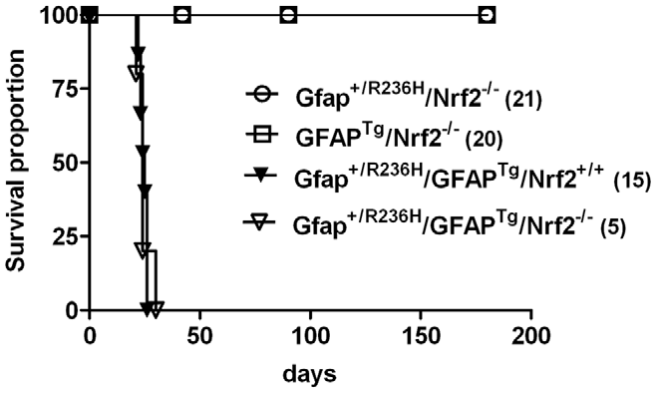
Loss of Nrf2 in a lethal model of AxD does not affect survival. Double positive Gfap^+/R236H^/GFAP^Tg^ mice that die at approximately 25 days of age show no significant difference in survival with loss of Nrf2 (Gfap^+/R236H^/GFAP^Tg^/Nrf2^+/+^, N = 15; Gfap^+/R236H^/GFAP^Tg^/Nrf2^−/−^, N = 5, Log-rank (Mantel-Cox) Test). For GFAP^Tg^/Nrf2^−/−^ and Gfap^+/R236H^/Nrf2^−/−^ mice, animals were euthanized at 6 wk and 3 mo time points for various experiments: for GFAP^Tg^/Nrf2^−/−^ (6 wks n = 20, 3 mos n = 12, 6 mos n = 2) for Gfap^+/R236H^/Nrf2^−/−^ (6 wks n = 21, 3 mos n = 14, 6 mos n = 7).

In our most severe A×D model, mice with a combination of mutant Gfap and over-expression of wild-type human GFAP (Gfap^+/R236H^/GFAP^Tg^) die apparently from seizures at about 25 days of age (Figure 7). To further assess the role of Nrf2 in AxD, we crossed the Gfap mutation together with the GFAP transgene into the Nrf2 null background. We anticipated that the survival of these mice would indicate whether Nrf2 was providing a protective or harmful effect, however neither was the case: the loss of Nrf2 in this combination had no effect on the lifespan of the mice (Figure 7).

## Discussion

We have two main interests for pursuing genetic modifiers in the context of AxD, one mechanistic and one therapeutic. From the mechanistic standpoint, removing protective modifiers in the mouse may worsen pathology and help explain differences between the human and mouse phenotypes. Such modifiers would offer insights into the progression of cellular dysfunction. In addition, the identification of protective modifiers could ultimately prove to have therapeutic potential for AxD and other neurodegenerative disorders with astropathology.

Nrf2 is a member of the CNC (cap-n-collar) subfamily of basic leucine zipper transcription factors, originally identified in the erythroid system in studies of globin DNAse I hypersensitive sites, but now known to be widely expressed in multiple tissues [33]. Normally Nrf2 is inactive and confined to the cytoplasm through binding of the Kelch-like protein, Keap1, ubiquitinated and degraded. In response to a wide variety of stresses, and especially oxidative stress, Nrf2 dissociates from Keap1 and translocates to the nucleus, where it binds to a short antioxidant response element (ARE) found in the promoters of a number of stress response genes such as Nqo1, glutathione-S-transferase, and heme oxygenase-1, thus activating their expression [36]. Alternatively Nrf2 can be targeted for proteasomal degradation through phosphorylation of the Neh6 domain by GSK-3β or other kinases [20], [21]. The closely related Nrf1 also binds to the ARE, and along with other factors such as AP1, SP1, and C/EBP may contribute to the basal levels of expression of these target genes. However, the marked up-regulation that occurs via the ARE in response to stress appears to be largely, if not entirely, mediated through Nrf2 [37]. In this light, it is interesting that Nrf1-null mice die in utero due to defective erythropoiesis [38]. In contrast, Nrf2-null mice survive to adulthood, but are more susceptible to a variety of insults, including MPTP and rotenone [23], 6-hydroxydopamine [39], kainic acid [40], 3-nitropropionic acid [24], [25], malonate [24], ischemia [22], and experimental autoimmune encephalomyelitis [41]. Conversely, increased expression of Nrf2, specifically in astrocytes, provides neuroprotection in a genetic mouse model of amyotrophic lateral sclerosis [26] and the MPTP mouse model of Parkinson’s disease [27].

We have found that there is marked induction of the ARE pathways in our mouse models of AxD [11], [12]. Given Nrf2’s common activation in a number of CNS disorders, the potential neuroprotective effects of enhancing expression, and the existence of several natural as well as synthetic inducers of Nrf2 activity that could have therapeutic applications [42], there is considerable appeal to further exploring its role in AxD and neurodegeneration.

Our findings however, show that loss of Nrf2 activation in AxD mice is surprisingly innocuous. Others have reported that Nrf2 null mice show white matter pathology with vacuolar degeneration and glial activation after 10 months of age [43]. We find no evidence of this in the younger mice focused on in our study (2–3 months of age), and loss of Nrf2 in GFAP^+/+^ mice did not cause a reduction in baseline levels for Nrf2 regulated genes as shown previously in Nrf2^−/−^ mice at 6 months of age [40]. In the AxD models examined: Gfap^+/R236H^ mutant, GFAP over-expressing transgenic (GFAP^Tg^), and double Gfap^+/R236H^/GFAP^Tg^ mice, with increasing severity respectively, loss of Nrf2 does not worsen the apparent phenotype, including life-span, body weight or the distribution of Rosenthal fibers in the brain. Astrocytes and microglia remain reactive, but further examination shows decreased expression of stress response genes that are not directly regulated by Nrf2. In hippocampus, expression of Gfap and the small heat shock protein Cryab, both of which are elevated during reactive gliosis, are reduced in Nrf2^−/−^ mice. Transcripts for the acute phase response protein ceruloplasmin are also reduced in olfactory bulb and hippocampal astrocytes, showing reduced reactivity in pathways that are not part of the phase II stress response.

Another common feature of neurodegenerative disease is the accumulation or re-distribution of iron. Normally, histological staining of ferric iron (Fe^3+^) in the brain shows prominent storage in oligodendrocytes, which is thought to reflect the energy requirements associated with maintaining myelin sheaths [44]. In Alzheimer’s and Parkinson’s diseases, however, aberrant iron storage occurs in cells other than oligodendrocytes [45]. In the mouse models of AxD, iron accumulates in astrocytes. This accumulation is markedly reduced with the loss of Nrf2. The genes encoding both heavy and light chains of the iron storage protein, ferritin, have ARE regulatory elements in their gene promoters [46], [47] (although these same genes are also controlled post-transcriptionally by the iron regulatory proteins, IRP1 and IRP2, in response to iron availability [48]). In AxD mice, transcription for the ferritin heavy chain gene (Fth1) is increased in olfactory bulb and hippocampus, regions with high Gfap and RF levels and marked ARE induction. Fth1 transcripts are then reduced back to wild-type levels with the knockout of Nrf2. Whether these changes in ferritin expression account for the diminished staining for iron observed histologically is not clear [49], since protein levels for either Fth1 or Ftl1 do not seem to change, regardless of Nrf2 activation.

Nrf2 also regulates the gene for p62 (sequestosome) [50], [51], the cargo receptor protein involved in targeting ubiquitinated proteins for autophagy [52]. p62 has been found to associate with several different cytoplasmic inclusion bodies including Rosenthal fibers [53]. In AxD mice p62 is elevated and associates with at least a portion of Gfap aggregates. In agreement with our previous report showing that mutant GFAP stimulates autophagy in vitro [14], these results suggest ubiquitinated Gfap aggregates [11] are targeted for autophagic degradation in vivo as well. In Nrf2 null mice with decreased p62 expression, one would expect less efficient targeting of Gfap aggregates for autophagy and further accumulation. Instead, we see a decrease in total Gfap protein, leaving the role of p62 in RF degradation unclear. Komatsu and others have recently reported harmful consequences resulting from Nrf2 activation in autophagy deficient mice, suggesting a balance between the two stress response pathways [54], [55]. Autophagy serves not only to remove damaged or pathogenic protein, but also functions in normal protein turnover [56]. By eliminating the barrage of antioxidant response proteins regulated by Nrf2, it is conceivable that autophagy becomes more efficient when contending with fewer proteins. Alternatively, reduced levels of p62 may simply reflect the decrease in Gfap accumulation.

Activation of microglia by injured and reactive astrocytes in AxD initiates cross talk between the two cell types via an exchange of cytokines and other secreted factors [12]. Reduced reactivity of astrocytes with the loss of Nrf2 may in part explain a decrease in the microglial response, but most likely the loss of Nrf2 in microglia themselves also contributes to this reduction. Diminished signaling between the two cell types may in turn further reduce glial reactivity.

We chose to focus on 2 brain regions with the highest levels of Gfap expression and ARE activation in our mutant AxD models: hippocampus and olfactory bulb. Although both regions showed decreased microglial activation and iron accumulation with loss of Nrf2, only hippocampus showed reduced Gfap and p62. Both regions have heterogenous astrocyte populations that could respond to the lack of Nrf2 in a cell specific manner [57]–[59].

The lack of detrimental effect with the loss of Nrf2 in our models of AxD is somewhat unexpected and suggests Nrf2 plays only a marginal role in the endogenous stress response, despite marked activation of Nrf2 regulated genes. Decreased expression of typical stress markers outside of the phase II detoxification response further complicates the role of Nrf2, and leads to a circular question of cause and consequence, perhaps fitting given the paradoxical role of astrocytes themselves in neuropathology. It will be of considerable interest to find whether further activation of Nrf2 is helpful or harmful in these same models.
